# A randomized, double-blinded, phase 2 trial of EDP1815, an oral immunomodulatory preparation of *Prevotella histicola*, in adults with mild-to-moderate plaque psoriasis

**DOI:** 10.3389/fmed.2024.1292406

**Published:** 2024-05-15

**Authors:** Benjamin D. Ehst, Bruce Strober, Andrew Blauvelt, Douglas Maslin, Debbie Macaro, Nancy Carpenter, Mark Bodmer, Duncan McHale

**Affiliations:** ^1^Oregon Medical Research Center, Portland, OR, United States; ^2^Yale University School of Medicine, New Haven, CT, United States; ^3^Central Connecticut Dermatology, Cromwell, CT, United States; ^4^Evelo Biosciences, Inc., Cambridge, MA, United States

**Keywords:** immunomodulation of skin, *Prevotella*, EDP1815, psoriasis, phase 2 clinical trial, small intestinal mucosa

## Abstract

**Background:**

Psoriasis is a chronic inflammatory skin disease. EDP1815 is an oral, gut-restricted preparation of non-live *Prevotella histicola*, the first of a new immunomodulatory therapeutic class targeting the small intestine to generate systemic anti-inflammatory responses.

**Objective:**

To evaluate safety and efficacy of EDP1815 in mild-to-moderate psoriasis in a proof-of-concept study.

**Methods:**

A phase 2, multicenter, randomized, double-blinded, placebo-controlled, parallel-group study with a 16-week treatment period and up to 24 weeks of follow-up. Participants were randomized to receive 1, 4, or 10 capsules daily.

**Results:**

EDP1815 was well tolerated with comparable rates of treatment-emergent adverse events to placebo, and no drug-related serious adverse events. Clinically meaningful responses to EDP1815, defined as at least 50% reduction in Psoriasis Area and Severity Index (PASI-50) at week 16, were observed in all 3 cohorts, statistically significant in the 1-capsule (29.7%; *P* = 0.048) and 4-capsule (31.9%; *P* = 0.022) groups, compared with placebo (12.1%). Among EDP1815-treated PASI-50 responders at week 16, 60% (18/30) maintained or improved off-treatment responses at week 40.

**Limitations:**

Continued off-treatment improvement past 16 weeks shows potential for greater therapeutic benefit that was not assessed.

**Conclusion:**

EDP1815 was well-tolerated with a placebo-like safety profile, and had meaningful efficacy outcomes in psoriasis, validating this novel immunomodulatory approach.

**Clinical trial registration:**

https://www.clinicaltrials.gov/search?term=NCT04603027, identifier NCT04603027.

## Introduction

**FIGURE 1:**
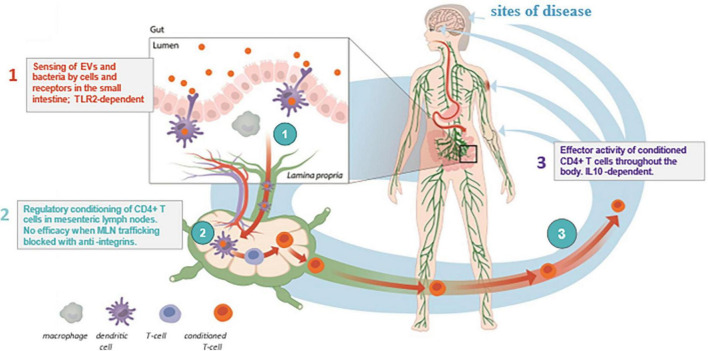
Immune-mediated diseases. Mechanism of Action of EDP1815. Depiction of the 3-step mechanism of action of EDP1815, which includes (1) sensing of EDP1815 by immune cells such as dendritic cells in the small intestine, (2) T cells trafficking through MLNs encounter enteric dendritic cells, which alter the CD4 + T cell to a regulatory phenotype, and then, (3) regulatory CD4 + T cells leave the mesenteric lymph node and enter the systemic circulation, where they migrate to sites of inflammation in peripheral tissues (2, 3). EV, extracellular vesicles; IL-10, interleukin-10; MLN, mesenteric lymph node; TLR-2, toll-like receptor 2.

EDP1815 is a non-live pharmaceutical preparation of a strain of *Prevotella histicola* (*P. histicola*) isolated from the duodenal mucosa of a single human donor (1). EDP1815 is gut-restricted after oral administration and does not colonize the gut nor impact the colonic microbiome. Instead, its mechanism of action harnesses the anti-inflammatory immune function of the small intestine, leading to inflammation resolution in the periphery (2). This is mediated by a 3-step process (Figure 1): (1) initial sensing during which structural motifs on the bacteria interact with pattern recognition receptors on immune cells, including dendritic cells in the gut; (2) cellular interactions in the mesenteric lymph nodes, which generate CD4^+^ T cells with a regulatory phenotype; and (3) these regulatory T cells leave the lymph nodes and circulate to resolve inflammation at sites remote from the gut (2, 3). Of note, oral administration of EDP1815 to mice leads to striking therapeutic efficacy in multiple *in vivo* models: delayed-type hypersensitivity, imiquimod-induced skin inflammation, fluorescein isothiocyanate cutaneous hypersensitivity, collagen-induced arthritis, and experimental autoimmune encephalomyelitis (1, 3–5). EDP1815 is the first agent specifically targeting a novel mechanism harnessing the mucosal immunology in the small intestine to generate regulatory CD4 + T cells that modulate multiple inflammatory pathways (2). It has the potential to address the unmet clinical needs of patients with a wide range of inflammatory diseases (2).

Psoriasis is a prevalent immune-mediated systemic inflammatory disease involving skin and joints (6, 7). It shares comorbidities with multiple other inflammatory and metabolic conditions (6, 8–12). Over 80% of psoriasis patients have mild or moderate disease, yet experience significantly diminished quality of life and psychosocial burden (8, 13, 14). Nearly half of these patients report treatment dissatisfaction and/or remain untreated despite available options (9, 15). Most agents approved for mild-to-moderate disease are topical and do not provide sustained effective relief due to poor adherence and short treatment courses (9, 13, 15). A safe, well-tolerated, effective, and affordable oral medication that addresses the underlying systemic inflammation driving psoriasis is needed for this underserved mild-to-moderate population. Here, we describe the safety and efficacy of EDP1815 in the treatment of adults with mild-to-moderate plaque psoriasis.

## Methods

### Study design

**FIGURE 2:**
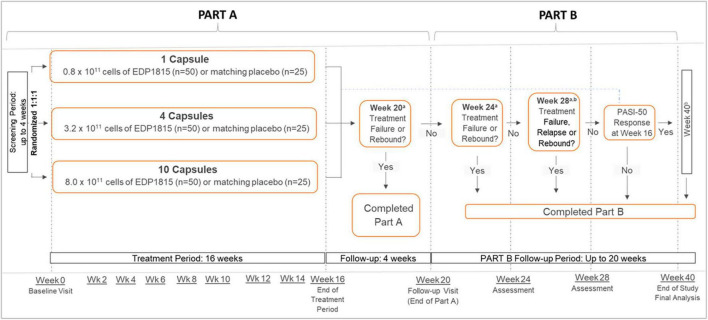
Psoriasis. Study design. All dosing was once daily. ^a^Treatment failure was defined as the initiation of an oral agent, biologic, or topical corticosteroid therapy for psoriasis. Relapse was defined as an increase in PASI to baseline level or greater, or the start of new psoriasis treatment, on or before week 40. Rebound was defined as an increase in PASI to ≥125% of baseline value, or an onset of new pustular/erythrodermic psoriasis, on or before the week 28 visit. ^b^Only participants who had achieved a PASI-50 or greater at week 16, referred to as PASI-50 responders, were followed from week 28 to week 40. PASI, Psoriasis Area and Severity Index; PASI-50, 50% reduction in PASI; wk, week.

Study EDP1815-201 (NCT04603027) was a multicenter, randomized, double-blinded, placebo-controlled, parallel-group study in adults with mild-to-moderate plaque psoriasis (Supplementary Appendix). The study comprised 2 parts (Figure 2): part A (4-week screening, 16-week treatment period, and 4-week follow up) and an optional part B (up to 20-week extended follow up without study drug or other psoriasis treatments). Depending on clinical response during part A, participants completed part B either at 28 or 40 weeks, or otherwise at the first occurrence of rebound or requirement for any rescue psoriasis medication. Rebound was defined as an increase in PASI of at least 125% from baseline or an onset of new pustular/erythrodermic lesions after stopping therapy (full definitions of terms are in the Supplementary Appendix).

Participants were randomized 1:1:1 to 3 parallel once-daily groups: a) 1 capsule of EDP1815 (0.8 × 10^11^ total cells daily) or placebo; or b) 4 capsules of EDP1815 (3.2 × 10^11^ total cells) or placebo; or c) 10 capsules of EDP1815 (8.0 × 10^11^ total cells) or placebo (Figure 2). Participants within each group were re-randomized 2:1 to receive either EDP1815 or placebo for 16 weeks. Randomization used a centrally-administered permuted block design.

The primary endpoint was the mean percentage change in PASI from baseline at week 16. Key secondary endpoints at week 16 included the achievement of PASI-50, PASI-75, and PASI-90, and the percent of participants with a Physician Global Assessment (PGA) score of 0 on a 6-point scale (0-5), and PGA score of 0 or 1 with a ≥ 2-point improvement from baseline. Other secondary endpoints were mean changes from baseline and responder analyses at weeks 4, 8, 12, 16, and 20, including Dermatology Life Quality Index (DLQI) and other patient-reported QoL outcomes. Secondary endpoints in part B measured the cumulative incidence of partial and complete relapse at weeks 20, 24, 28, and 40 among those who achieved PASI-50 at week 16, and cumulative incidence of rebound at weeks 20, 24, and 28 among participants who had at least 1 post-treatment PASI assessment and who had ≤ 125% increase from baseline in PASI at their last on-treatment visit. Safety and tolerability were evaluated based on adverse event (AE) reporting.

### Study population

Adults 18–70 years of age with mild-to-moderate plaque psoriasis for ≥ 6 months, defined as plaques covering a body surface area of ≥ 3% and ≤ 10%, a PASI of ≥ 6 and ≤ 15, and a PGA score of 2 or 3 were enrolled. Key exclusion criteria were psoriasis restricted to the scalp, palms, and soles, non-plaque psoriasis, or other skin conditions that would interfere with evaluation of response. Topical unmedicated emollients and low-potency topical corticosteroids were permitted if being used prior to study entry and continued in the same manner. Topical or systemic rescue therapy for psoriasis was not permitted. Initiation of a new psoriasis treatment during the study was considered a treatment failure.

### Statistical analysis

The target sample size was 225 subjects with at least 4 weeks of data. Participants who withdrew from the study prior to week 4 were replaced. The primary efficacy estimand used a while-on-treatment strategy, including all participants in the modified intent-to-treat population (participants who were randomized and received at least 1 dose of study treatment), but excluding any data collected more than 4 days after last dose. Missing data were accounted for using mixed models for repeated measures (MMRM). Safety estimands included all participants who received at least 1 dose of study medication.

A model-based probability inference framework was used for primary efficacy analyses. Bayesian MMRM were used for the analysis of continuous data, including the primary endpoint. Posterior estimates and 95% credible intervals (CrI) for the difference between each active capsule cohort and placebo were quantified.

### Ethical considerations

This study was performed in accordance with the Declaration of Helsinki, the International Conference on Harmonization Good Clinical Practice Guideline, and all applicable country and local regulations. Participants provided written informed consent before trial entry or performing non-routine procedures.

## Results

### Study participants

In this study, 411 participants were screened from which 249 were randomized 1:1:1 into 3 capsule-number cohorts, then re-randomized 2:1 to receive either EDP1815 or placebo. Ten randomized participants were later found to have failed entry criteria. Of the 249 randomized and treated participants in the modified intent-to-treat (mITT) population, 186 (74.7%) completed part A. 124 (66.7%) entered and 109 (58.6%) completed part B (Supplementary Figure 1). Nearly all (99.6%) participants were ≥ 80% compliant, with a median of 100% compliance in each group. One participant in each of the four treatment groups did not have at least one post-baseline efficacy assessment and thus were excluded from the efficacy models; 55 other participants did not have on-treatment week 16 efficacy assessments, but were included in the repeated measures analyses up to the point of treatment discontinuation. Most participants were White (98.8%) males (63.1%) with moderate psoriasis per baseline PGA score (62.2%). Baseline demographics and disease characteristics were similar across treatment groups (Supplementary Table 1).

### Efficacy of EDP1815

**FIGURE 3:**
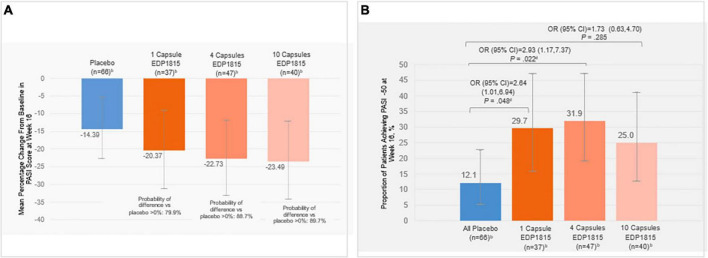
**(A)** Posterior mean percentage change in PASI from baseline at week 16^a^, **(B)** Proportion of patients achieving PASI-50 at week 16^c^. ^a^Error bars indicate 95% HPD credibility intervals for posterior mean percentage change in PASI. Posterior probabilities of superiority of the treatment difference between EDP1815 groups compared to placebo ranged from approximately 80–90% across the prespecified analyses and cohorts. ^b^Number of participants with analyzable data at week 16. ^c^Error bars indicate 95% CI for the proportion of patients achieving PASI-50 at week 16. ^d^Denotes statistical significance (*P* < 0.05). Percent change from baseline in PASI at each visit was calculated as: 100×(PASI at visit – baseline PASI)/baseline PASI. CI, confidence interval; HPD, high posterior density; OR, odds ratio; PASI, Psoriasis Area and Severity Index; PASI-50, 50% reduction in PASI.

The primary endpoint of mean percent change in PASI from baseline at week 16 showed a numerical improvement for all EDP1815-treated groups compared with placebo (Figure 3A). Posterior probabilities of superiority of the EDP1815 groups compared to placebo with a probability of difference > 0% were 79.9%, 88.7%, and 89.7% in the respective 1-, 4-, and 10-capsule EDP1815 groups.

At week 16, PASI-50 was achieved by 12% of placebo-treated patients, compared with 29.7% (1 capsule, *P* = 0.048), 31.9% (4 capsules, *P* = 0.022), and 25.0% (10 capsules, *P* = 0.285) of EDP1815-treated patients (Figure 3B). Given the lack of evidence for a difference in capsule number response, an *ad hoc* analysis was performed combining the treatment groups, with an overall response rate of 29.0%, (*P* = 0.027).

A numerically higher proportion of EDP1815-treated participants achieved PASI-75 and PASI-90 at week 16 compared with those receiving placebo (Supplementary Table 2). Similarly, a higher proportion of EDP1815-treated participants achieved PGA scores of 0 or 1 with a ≥ 2-point improvement from baseline compared to placebo (Supplementary Table 2).

*Post hoc* analysis showed that a PGA score of 0 or 1 was achieved by 20.2% of pooled EDP1815-treated participants compared with 9.1% of those in the placebo group (*P* = 0.048) (Supplementary Table 2). Non-statistically significant numerical trends were observed in favor of EDP1815 over placebo in other secondary endpoints, including participant-reported outcomes and/or QoL endpoints.

Part B participants who were followed for a further six months off drug comprised 30 participants from the EDP1815 treatment groups and 8 participants from the placebo group who had reached at least PASI-50 at week 16. After cessation of dosing, 18/30 (60%) of EDP1815-treated participants remained at PASI-50 or better at week 40. Half (5/10) of EDP1815-treated PASI-75 responders at week 16 maintained response at week 40. In addition, 9/20 (45%) participants who had achieved PASI-50, but not PASI-75, at week 16 showed increased responses to PASI-75 or greater on or before week 40. Finally, 3/38 (7.9%) participants in the pooled EDP1815 group achieved PASI-100 while off all therapy during part B, despite no study participants reaching PASI-100 in the 16-week treatment phase. No placebo patients achieved PASI-100.

**FIGURE 4:**
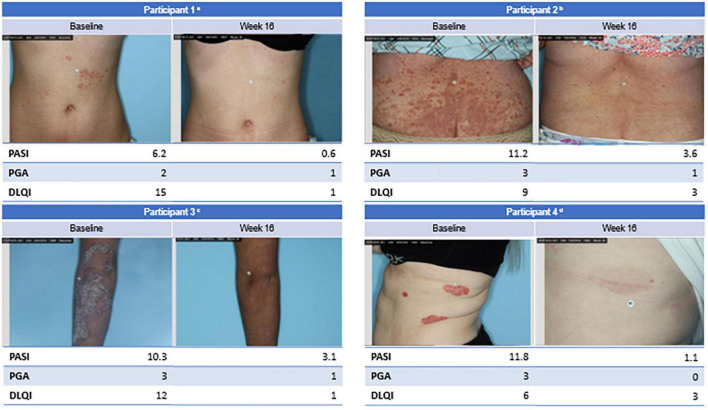
Clinical imagery of select participants. ^a^Female participant with mild disease impacting quality of life since childhood in the 4 capsules/day EDP1815 group achieved PASI-90 at week 16. ^b^Female participant with recently diagnosed moderate-to-severe diffuse, small-plaque psoriasis in the 1 capsule/day EDP1815 group achieved PASI-50 at week 16. ^c^Female participant with moderate-to-severe thick, large plaque psoriasis diagnosed 10 years ago in the 4 capsules/day EDP1815 group achieved PASI-50 at week 16. ^d^Female participant with moderate-to-severe large plaque psoriasis diagnosed 20 years ago in the 1 capsule/day EDP1815 group achieved PASI-90 at week 16. BMI, body mass index; DLQI, Dermatology Life Quality Index; PASI, Psoriasis Area and Severity Index; PASI-50, 50% reduction in PASI; PASI-90, 90% reduction in PASI; PGA, Physician Global Assessment.

Psoriatic plaques from 4 representative participants who responded to EDP1815 are shown at baseline and week 16 (Figure 4). Parallel improvements in PASI, PGA, and DLQI scores were observed in these participants.

### Safety and tolerability

Treatment-emergent adverse events (TEAEs) occurred in 45 participants (54.2%) who received placebo and 97 participants (58.4%) who received EDP1815 (Table 1). Most TEAEs were mild or moderate in severity and not related to study treatment, with comparable rates between treatment groups and placebo. The most commonly reported TEAEs were also comparable between EDP1815 and placebo (Supplementary Table 3). No EDP1815-related AEs emerged during part B of the study.

**TABLE 1:** Overview of related treatment-emergent adverse events in part A of study (safety population).

Description	All Placebo (*N* = 83) n (%)	1 Capsule EDP1815 (*N* = 56) n (%)	4 Capsule EDP1815 (*N* = 55) n (%)	10 Capsule EDP1815 (*N* = 55) n (%)	All EDP1815 (*N* = 166) n (%)
Any TEAE^a^	45 (54.2)	30 (53.6)	31 (56.4)	36 (65.5)	97 (58.4)
Any related TEAE^b^	14 (16.9)	6 (10.7)	12 (21.8)	14 (25.5)	32 (19.3)
Any related TEAE of CTCAE grade ≥ 2	6 (7.2)	3 (5.4)	4 (7.3)	6 (10.9)	13 (7.8)
Any related TEAE of CTCAE grade ≥ 3	0	0	0	1 (1.8)	1 (0.6)
Any related TEAE of CTCAE grade ≥ 4	0	0	0	0	0
Any related fatal TEAE	0	0	0	0	0
Any related serious TEAE	0	0	0	0	0
Any related TEAE leading to permanent discontinuation of study drug^c^	2 (2.4)	0	0	1 (1.8)	1 (0.6)
Any related TEAE leading to withdrawal from the study^c^	1 (1.2)	0	0	0	0

^a^A TEAE is defined as an adverse event with onset date and time on or after the date and time of the first dose of study drug. Adverse events are graded using CTCAE criteria Version 5.0.

^b^Related TEAEs are those with possible, probable, or definite relationship to study drug, or where relationship is missing.

^c^Treatment discontinuation and study withdrawal due to worsening of psoriasis were reported as lack of efficacy or treatment failure. Only events of new or clinically significant worsening symptoms of psoriasis, or a new form of psoriasis, were reported as adverse events. CTCAE, Common Terminology Criteria for Adverse Events; TEAE, treatment-emergent adverse event.

Six participants discontinued the study due to TEAEs: 3 participants (3.6%) in the pooled placebo groups and 1 participant (1.8%) in each of the 3 EDP1815 groups. There were no serious adverse events (SAEs) related to the study drug in either parts A or B. Safety data were comparable between all groups of EDP1815 and placebo for up to 24 weeks after cessation of dosing.

There was no evidence of increased risk of rebound at week 28 after EDP1815 treatment. In the pooled placebo group, 7/64 participants (10.9%) experienced rebound compared to 9/125 (7.2%) in the pooled EDP1815 group

## Discussion

EDP1815 is a novel oral immunomodulatory agent with a completely new mechanism of action for the management of psoriasis, acting via the small intestinal axis to safely bring about resolution of systemic inflammation (Figure 1). EDP1815 was well-tolerated, with incidences of TEAEs similar to placebo. The primary efficacy analysis demonstrated superiority of EDP1815 to placebo in each cohort with a posterior probability of between 80 and 90%. A significantly greater proportion of participants achieved PASI-50 or PGA-0/1 at week 16 in the EDP1815-treated group than in the placebo group. This is the first report of a phase 2 clinical trial investigating this particular type of mechanism of action.

Interestingly, increasing the number of capsules taken daily did not reproduce the dose response clearly seen in pre-clinical studies. This is likely due to the different methods of altering dose in mice vs. humans, which lead to differential effects on pharmacokinetics in the continuously flowing environment of the intestines. Specifically in mice, the dose is increased by changing the concentration of drug in a fixed volume, leading to a higher drug concentration at the target site in the small intestine. By contrast in humans, the dose was increased by the number of capsules of drug administered. However, due to the variable kinetics of passage from the stomach into the small intestine and breakdown of the polymer coating on the capsules, administration of more capsules in humans would not necessarily lead to an increased drug concentration at any given point. Indeed, increasing the total drug administered through increased capsule number apparently did not increase the pharmacodynamic response. Potential methods to increase the pharmacodynamic response in man include increasing drug concentration within a single capsule, and developing a capsule that releases EDP1815 rapidly after exiting the stomach – causing a bolus of drug at the target site.

The majority of participants who achieved PASI-50 during the 16-week treatment period maintained PASI responses during the off-treatment follow-up period. The median time to loss-of-response was greater than the 6-month follow-up period. Furthermore, several PASI-50 responders at week 16 had increased efficacy responses to PASI-75 or greater, and 3 participants eventually achieved PASI-100 during the extended follow up period. It appears the clinical benefit of EDP1815 continued to accumulate beyond the 16-week treatment period. This is consistent with the mechanism of action of EDP1815 observed in preclinical models, suggesting an extended effect due to the generation and persistence of circulating regulatory CD4 + T cells (2, 3).

Due to the placebo-like safety data, the benefit-risk profile observed supports further investigation of EDP1815 in mild-to-moderate psoriasis, either as monotherapy or in combination with other therapies, including topical medications. A placebo-like safety profile together with oral delivery are key factors for both clinicians and patients when selecting psoriasis treatments (16).

This efficacy of EDP1815 could be further investigated by extending the treatment period to 24 weeks and using more sensitive endpoints in this population. Mean change on a non-linear scale such as PASI is less discriminating than a threshold responder analysis, since non-linear scales are relatively insensitive to change in milder disease (17–19). Treatment goals such as clear or almost clear skin (PGA-0/1) may be more sensitive and clinically relevant for demonstrating treatment effect.

In conclusion, the results reported here demonstrate that EDP1815 treatment led to clinically meaningful improvements in adult patients with mild-to-moderate psoriasis with a placebo-like safety and tolerability profile. This is the first phase 2 study to establish that a non-live preparation of a single gut-restricted bacterial strain can be delivered orally to the intestine to modulate inflammation in the periphery. These findings support the further development of EDP1815 for the treatment of psoriasis and other inflammatory conditions.

It has long been known that exposure to foreign matter in the gut prevents *systemic* inflammation to that matter (20). The outcome of this study provides proof of concept that this biologic property of intestinal mucosal immunology may be harnessed with a new type of oral, effective, safe and well-tolerated medicine, of which EDP1815 is the first example.

## Data availability statement

The original contributions presented in this study are included in the article/Supplementary material, further inquiries can be directed to the corresponding author.

## Ethics statement

The studies involving humans were approved by the Hungary Medical Research Council Ethics Committee for Clinical Pharmacology, Budapest, Zrínyi u. 3., H-1051, Budapest, Hungary Poland The Ethics Committee at the Regional Chamber of Physicians and Dentists, 80-204 Gdańsk, ul. Śniadeckich 33, Gdansk, Poland United Kingdom Health Research Authority, East Midlands - Nottingham 2 Research Ethics Committee The Old Chapel, Royal Standard Place, Nottingham, NG1 6FS, United Kingdom United States Advarra, 6940 Columbia Gateway Dr., Suite 110, Columbia, MD 21046, United States. The studies were conducted in accordance with the local legislation and institutional requirements. The participants provided their written informed consent to participate in this study.

## Author contributions

AB: Investigation, Writing – review and editing. BE: Investigation, Writing – review and editing. BS: Conceptualization, Writing – review and editing. DeM: Conceptualization, Investigation, Writing – review and editing. DoM: Conceptualization, Data curation, Formal analysis, Investigation, Methodology, Writing – original draft, Writing – review and editing. DuM: Conceptualization, Investigation, Methodology, Supervision, Writing – review and editing. MB: Conceptualization, Supervision, Writing – review and editing. NC: Conceptualization, Data curation, Formal analysis, Methodology, Writing – review and editing.
