# Supplementary material for: A randomized, double-blinded, phase 2 trial of EDP1815, an oral immunomodulatory preparation of Prevotella histicola, in adults with mild-to-moderate plaque psoriasis
Source: Front Med (Lausanne). 2024 May 15;11:1292406. doi: 10.3389/fmed.2024.1292406 (PMC11133679; doi:10.3389/fmed.2024.1292406)
Supplement: Supplementary file 1 [file Data_Sheet_1.pdf]

MENDELEY SUPPLEMENTAL FIGURES AND TABLES

Supplementary Appendix

Definition of Terms

Term	Definition
Partial relapse	Loss of PASI-50 response after cessation of study treatment
PASI	Psoriasis Area and Severity Index
PASI-50 response	≥50% reduction from baseline in PASI
PASI-75 response	≥75% reduction from baseline in PASI
PASI-90 response	≥90% reduction from baseline in PASI
PASI-100 response	100% reduction from baseline in PASI
Rebound	Increase in PASI to 125% of baseline value or greater, or onset of new pustular/erythrodermic psoriasis on or before the week 28 visit (excluding subjects with 125% increase at the end of treatment: rebound can be only achieved by subjects with the last on-treatment visit PASI less than 125% of baseline)
Relapse	Increase in PASI to baseline value or greater, or participant begins a new treatment for psoriasis
Treatment failure	Initiation of an oral agent, biologic, or intermediate or high-potency topical therapy for psoriasis

Study Centers and Countries

This study was conducted at 29 study centers in the following 4 countries: United States (8 study centers), United Kingdom (11 study centers), Poland (8 study centers), and Hungary (2 study centers).

Principal Investigators

Hungary

Aniko Maria Dobribanne Fekete, AES - DRS - Synexus Zalaegerszeg Magyarország Egészségügyi Kft, Zalaegerszeg, Hungary

Mónika Sápy, AES - DRS - Synexus Budapest - Magyarország Egészségügyi Szolgáltató Kft, Budapest, Hungary

Poland

Ryszard Galus, AES - DRS - Synexus Polska Sp. z o.o. Oddzial w Warszawie, Warszawa, Poland

Anna Janowicz, AES - DRS - Synexus Polska Sp. z o.o. Oddzial w Czestochowie, Czestochowa, Poland

Tomasz Kolodziej, AES - DRS - Synexus Polska Sp. z o.o. Oddzial we Wroclawiu, Wroclaw, Poland

Joanna Renczynska-Matysko, AES - DRS - Synexus Polska Sp. z o.o. Oddzial w Gdansku, Gdansk, Poland

Joanna Sieniawska, AES - DRS - Synexus Polska Sp. Z o.o. Oddzial w Lodzi, Lodz, Poland

Rafal Slugocki, AES - DRS - Synexus Polska Sp. z o.o. Oddzial w Gdyni, Gdynia, Poland

Santa Vanaga-Besser, AES - DRS - Synexus Polska Sp. z o.o. Oddzial w Katowicach, Katoice, Poland

Grażyna Wolnik-Trzeciak, AES - DRS - Synexus Polska Sp. z o.o. Oddzial w Poznaniu, Poznan, Poland

United Kingdom

Marianne Elizabeth Viljoen, AES - DRS - NW Consortium Merseyside, Liverpool, United Kingdom

Marianne Elizabeth Viljoen, AES - DRS - AES - DRS - NW Consortium Manchester, Manchester, United Kingdom

Marianne Elizabeth Viljoen, AES - DRS - NW Consortium Lancashire, Chorley, United Kingdom

Naimat (Naimatullah) Khan, Medicines Evaluation Unit, Manchester, United Kingdom

Kim-Claire Tabner, MAC Clinical Research, Cannock, United Kingdom

Abimbola Babajide, MAC Clinical Research, Lancashire, Blackpool, United Kingdom

Sandra Connell, MAC Clinical Research, Teeside, United Kingdom

Sam Leving, MAC Clinical Research, Leeds, United Kingdom

Imrozia Munsoor, AES - DRS - Synexus Scotland Clinical Research Centre, Lanarkshire, Bellshill, United Kingdom

Hawys Thomas, AES - DRS - Synexus Wales Clinical Research Centre, Cardiff, United Kingdom

Hawys Thomas, AES - DRS - Synexus Thames Valley Clinical Research Centre, Reading, United Kingdom

United States

Benjamin Ehst, Oregon Medical Research Center PC, Portland, Oregon, United States

Kenneth Stein, AES - Synexus Clinical Research US, Inc. - Santa Rosa, Santa Rosa, California, United States

Paul Nugent, AES - DRS - Synexus Clinical Research US, Inc. – Cincinnati, Cincinnati, Ohio, United States

Richard Sturtz, AES - DRS - Synexus Clinical Research US, Inc. – Anderson, Anderson, South Carolina, United States

Jewel White, AES - DRS - Synexus Clinical Research US, Inc. - The Villages, The Villages, Florida, United States

Robert Meyer, AES - DRS - Synexus Clinical Research US, Inc. – Orlando, Orlando, Florida, United States

Seth Forman, ForCare Clinical Research - CenExcel FCR – PPDS, Tampa, Florida, United States

Linda Murray, AES - DRS - Synexus Clinical Research US, Inc. - St. Petersburg, Pinellas Park, Florida, United States

Institutional Review Boards/Ethics Committees

Hungary

Medical Research Council Ethics Committee for Clinical Pharmacology, Budapest, Zrínyi u. 3., H-1051, Budapest, Hungary

Poland

The Ethics Committee at the Regional Chamber of Physicians and Dentists, 80-204 Gdańsk, ul. Śniadeckich 33, Gdansk, Poland

United Kingdom

Health Research Authority, East Midlands - Nottingham 2 Research Ethics Committee

The Old Chapel, Royal Standard Place, Nottingham, NG1 6FS, United Kingdom

United States

Advarra, 6940 Columbia Gateway Dr., Suite 110, Columbia, MD 21046, United States

Additional Parameters Considered in Statistical Analysis

Sample size (N=225) was calculated to ensure at least 80% power at the 5% 2-sided significance level to detect a difference of 20% between the active and placebo groups within each cohort for percent change in PASI from baseline at week 16. Pooled standard deviation across all doses was assumed to be 25% and the drop-out rate between week 4 and week 16 was assumed to be 15%. The 3 placebo groups were examined for systematic differences between them and as none were found, all placebo groups were pooled for pairwise comparisons between each active dose and placebo.

Response endpoints were analyzed using a frequentist generalized linear mixed model with a logit link. Descriptive statistics were also used to summarize all endpoints by treatment group. All analyses were conducted using SAS software (SAS Institute, Inc, Cary, North Carolina) Version 9.4.

Mendeley Supplemental Figure 1. Participant Disposition

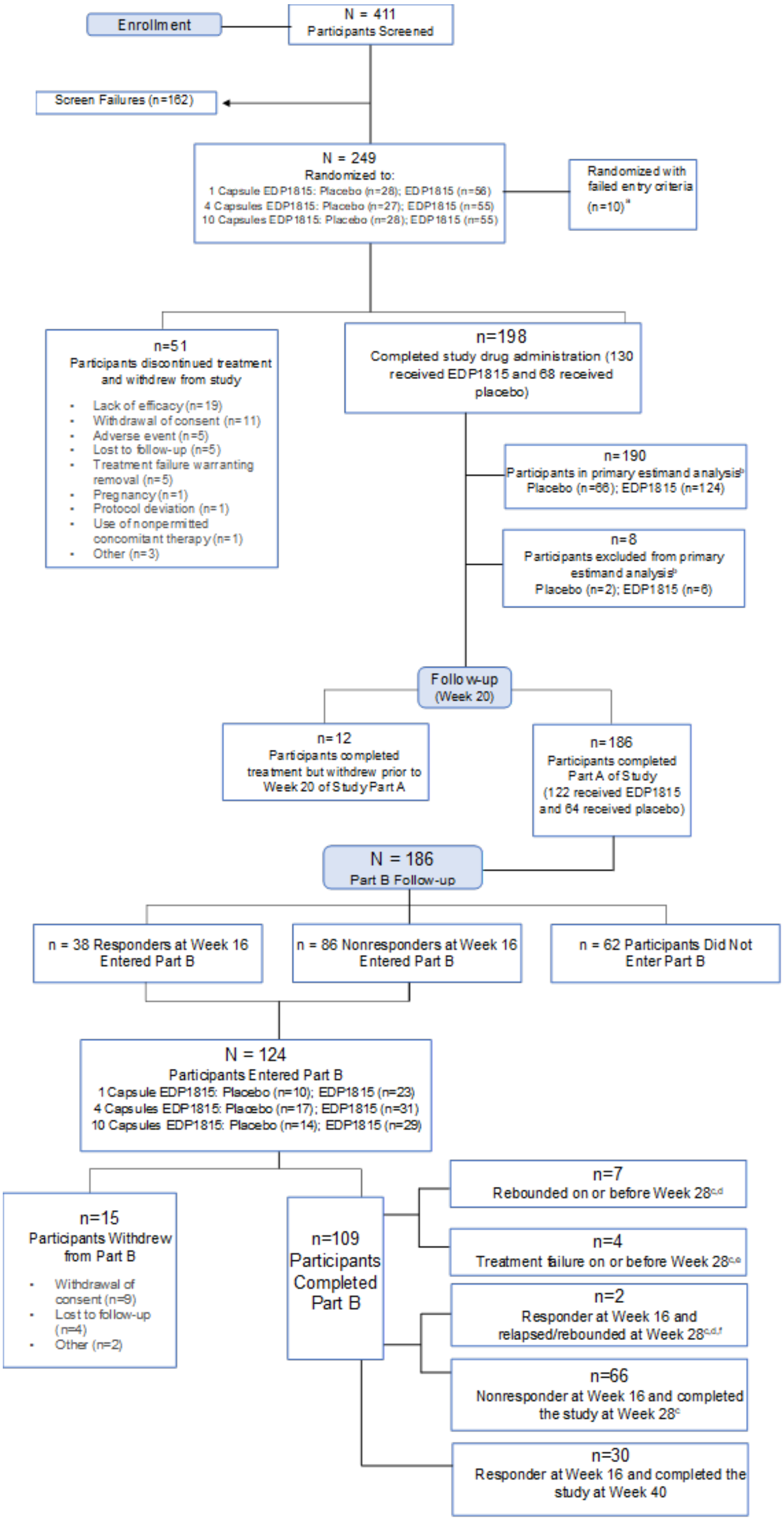

[a]Failed entry criteria for randomized participants included contraception criteria, having received systemic or topical treatment for psoriasis, chronic inflammatory disease, and recent blood donation.

[b]Primary efficacy estimand analysis included participants with week 16 data available within 4 days after end of treatment and excluded those without.

[c]Participants were considered to have completed the study.

[d]Rebound was defined as an increase in PASI to ≥125% of baseline value, or an onset of new pustular/erythrodermic psoriasis, on or before the week 28 visit.

[e]Treatment failure was defined as the initiation of an oral agent, biologic, or intermediate or high-potency topical therapy for psoriasis.

[f]Relapse was defined as an increase in PASI to baseline level or greater, or the start of new psoriasis treatment, on or before week 40.

Mendeley Supplemental Table I. Summary of Baseline Demographics

	All (n=249)	Pooled Placebo (n=83)	1 Capsule EDP1815 (N=56) n (%)	4 Capsules EDP1815 (N=55) n (%)	10 Capsules EDP1815 (N=55) n (%)
Age, mean years	44	44.4	44.5	41.2	45.5
Female (%)	36.9	39.8	39.3	43.6	23.6
White (%)	98.8	99.4	100	98.2	97.6
BMI, mean kg/m^2	29.7	30.2	29.9	28.5	29.7
Duration of plaque psoriasis, mean years	18.6	17.8	18.6	18.0	20.6
PGA 2 (%)	37.8	34.9	37.5	45.5	34.6
PGA 3 (%)	62.2	65.1	62.5	54.5	65.4
PASI mean (%)	8.5	8.7	8.4	8.7	8.3
BSA mean (%)	7.3	7.3	7.4	7.3	7.1

BMI, body mass index; BSA, body surface area; DLQI, Dermatology Life Quality Index; kg, kilogram; m, meter; PASI, Psoriasis Area and Severity Index; PGA, Physician Global Assessment.

Mendeley Supplemental Table II. Summary of Key Secondary and Post Hoc Outcomes at Week 16

	Placebo	1 Capsule EDP1815		4 Capsules EDP1815		10 Capsules EDP1815	
	Responders	Responders	OR vs. Placebo[a]	Responders	OR vs. Placebo[a]	Responders	OR vs. Placebo[a]
**Key Secondary Outcomes**							
**PASI-50, n/N (%)**	8/66 (12.1)	11/37 (29.7)	2.64	15/47 (31.9)	2.93	10/40 (25.0)	1.73
**PASI-75, n/N (%)**	2/66 (3.0)	2/37 (5.4)	N/A	4/47 (8.5)	N/A	4/40 (10.0)	N/A
**PASI-90, n/N (%)**	0/66 (0)	0/37 (0)	N/A	1/47 (2.1)	N/A	2/40 (5.0)	N/A
**Achievement of PGA 0 or 1 with a ≥2-point improvement from baseline, n/N (%)**	4/66 (6.1)	4/37 (10.8)	2.14	5/47 (10.6)	1.73	5/40 (12.5)	1.89
**Key Post Hoc Outcome**							
**Achievement of PGA 0 or 1, n/N (%)**	6/66 (9.1)	8/33 (24.3)	N/A	10/47 (21.3)	N/A	6/40 (15.0)	N/A

[a]Odds ratios calculated using a generalized linear mixed model with a logit link. PASI-75 and PASI-90 endpoints were summarized only.

N/A, not available; OR, odds ratio; PASI, Psoriasis Area and Severity Index; PGA, physician's global assessment.

Mendeley Supplemental Table III. Summary of Related TEAEs Reported by at Least 2% of Participants in Any Treatment Group

**System Organ Class** Preferred Term	**All Placebo (N=83) n (%), E**	**1 Capsule EDP1815 (N=56) n (%), E**	**4 Capsules EDP1815 (N=55) n (%), E**	**10 Capsules EDP1815 (N=55) n (%), E**	**All EDP1815 (N=166) n (%), E**
**Any related event[a]**	14 (16.9%), 32	6 (10.7%), 17	12 (21.8%), 31	14 (25.5%), 27	32 (19.3%), 75
**Nervous system disorders**					
Headache	3 (3.6%), 4	2 (3.6%), 2	0	1 (1.8%), 1	3 (1.8%), 3
**Gastrointestinal disorders**					
Diarrhea	3 (3.6%), 4	2 (3.6%), 2	1 (1.8%), 1	3 (5.5%), 3	6 (3.6%), 6
Dyspepsia	2 (2.4%), 2	0	4 (7.3%), 7	2 (3.6%), 2	6 (3.6%), 9
Abdominal pain	1 (1.2%), 1	1 (1.8%), 2	2 (3.6%), 3	1 (1.8%), 1	4 (2.4%), 6
Flatulence	0	0	3 (5.5%), 3	1 (1.8%), 1	4 (2.4%), 4
Nausea	1 (1.2%), 1	1 (1.8%), 2	0	2 (3.6%), 2	3 (1.8%), 4
Abdominal distention	1 (1.2%), 2	0	0	2 (3.6%), 2	2 (1.2%), 2
**Skin and subcutaneous tissue disorders**					
Rash	0	1 (1.8%), 1	0	2 (3.6%), 2	3 (1.8%), 3
Pruritus	4 (4.8%), 5	0	1 (1.8%), 1	0	1 (0.6%), 1
**Investigations**					
C-reactive protein increased	0	0	0	2 (3.6%), 2	2 (1.2%), 2

[a]Related TEAEs are those with possible, probable, or definite relationship to study drug, or where relationship is missing.

E, events; TEAE, treatment-emergent adverse event.
